# Clinicopathological Analysis of 1,000 Cases of Head and Neck Squamous Cell Carcinoma With Special Emphasis on Histological Variants and p16 Expression

**DOI:** 10.7759/cureus.117616

**Published:** 2026-10-07

**Authors:** R P Sushma Kumari, M Mamatha, Naval Kishore Bajaj, Syed Allauddin Khadri

**Affiliations:** 1 Pathology, Osmania Medical College, Hyderabad, IND; 2 Pathology, Taibah University, Madinah, SAU

**Keywords:** cervical lymph node metastasis, clinicopathological profile, head and neck squamous cell carcinoma, histological variants, human papillomavirus, p16 immunohistochemistry

## Abstract

Background: Head and neck squamous cell carcinoma (HNSCC) remains a major clinical and pathological concern in India. Its presentation varies by anatomical site and histological pattern, and recognizing uncommon variants, along with the appropriate interpretation of p16 expression, may provide useful diagnostic information.

Objective: This study aimed to describe the demographic and clinicopathological profile of 1,000 HNSCC cases, with particular attention to histological variants, cervical lymph node involvement, and p16 expression.

Materials and methods: This retrospective observational study included 1,000 histologically confirmed HNSCC cases diagnosed at a tertiary-care teaching hospital between January 2023 and December 2024. Age, sex, tumor site, histological grade, nodal status, histological variants, recurrence, and second primary malignancies were reviewed. p16 immunohistochemistry was performed in 109 cases with suitable tissue, including 50 conventional SCCs and 59 histological variants. Strong, diffuse nuclear and cytoplasmic staining in more than 70% of tumor cells was considered positive.

Results: The cohort included 832 men and 168 women, with most patients aged 51-70 years. The oral tongue was the most frequent individual site (24.7%), while supraglottic and glottic tumors together accounted for 29.4% of cases. Well-differentiated SCC was the predominant grade (71.0%). Cervical lymph node metastasis was present in 147 patients (14.7%), with the highest within-grade frequency in moderately differentiated SCC (20.4%); the association between histological grade and nodal metastasis was significant (χ² = 9.89, p = 0.007). Of the 59 histological variants, 32 (54.2%) were p16 positive, with papillary SCC accounting for most positive cases. Of the 50 conventional SCCs, 20 (40.0%) showed p16 positivity. Twenty-five patients had second primary malignancies, and 16 had recurrence.

Conclusions: This large single-center series demonstrates the clinicopathological diversity of HNSCC in an Indian tertiary-care setting. Histological variant recognition and assessment of nodal involvement remain important components of pathological evaluation. p16 immunohistochemistry may provide useful supplementary information in selected cases, but its expression should be interpreted as a surrogate marker rather than definitive evidence of HPV-driven disease without molecular confirmation.

## Introduction

Head and neck cancers remain an important cause of cancer-related morbidity and mortality worldwide. According to GLOBOCAN 2022 estimates from the International Agency for Research on Cancer, cancers of the lip and oral cavity accounted for 389,846 new cases and 188,438 deaths globally. During the same period, laryngeal cancer accounted for 189,191 new cases and 103,359 deaths, while approximately 120,434 cases of nasopharyngeal cancer and 86,257 cases of hypopharyngeal cancer were reported worldwide [1]. The burden is particularly relevant to India, where lip and oral cavity cancer was the most frequently diagnosed cancer among men in 2022, with an estimated 107,812 new cases [1]. More than 90% of malignant tumors arising from the mucosal surfaces of the oral cavity, pharynx, and larynx are squamous cell carcinomas (SCCs) [2]. Despite improvements in surgery, radiotherapy, and systemic treatment, late presentation, locoregional recurrence, and second primary malignancies continue to influence the clinical course of head and neck SCC (HNSCC) [3].

The distribution and etiological profile of HNSCC vary considerably across geographical regions. In South and Southeast Asia, including India, tobacco smoking, smokeless tobacco, use of areca nut and betel quid, and alcohol consumption are well-established contributors to the high burden of oral and other head and neck cancers [4,5]. These exposures are particularly relevant as background epidemiological factors in the Indian population, although they were not the primary focus of the present clinicopathological analysis. In contrast, the incidence of HPV-associated oropharyngeal SCC has increased substantially in several Western populations, with HPV-16 accounting for most HPV-driven tumors at this site [6]. HPV-associated oropharyngeal carcinoma is now recognized as a biologically distinct disease with differences in treatment response and prognosis compared with conventional tobacco-related HNSCC [7].

Clinical presentation varies by anatomical site and disease extent. Patients may present with a persistent oral ulcer, dysphagia, change in voice, throat discomfort, or cervical lymphadenopathy, and some tumors are first diagnosed at an advanced stage [8]. Histopathological examination therefore remains central to diagnosis and tumor characterization. Although conventional SCC forms the largest group, HNSCC includes several less common histological variants with distinctive microscopic appearances and, in some instances, different biological behavior and prognostic implications [9]. Correct recognition of these variants is consequently important during routine pathological evaluation.

Assessment of HPV-related disease has also become increasingly relevant, particularly in oropharyngeal SCC. p16 immunohistochemistry is widely used as a practical surrogate marker of HPV-associated carcinogenesis; however, p16 overexpression does not by itself establish the presence of transcriptionally active HPV, especially in tumors arising outside the oropharynx. Its findings should therefore be interpreted together with tumor site, morphology, and, where clinically required, HPV-specific molecular testing [10]. This distinction is particularly important when examining p16 expression across different anatomical sites and uncommon histological variants.

Large single-center series describing the clinicopathological spectrum of HNSCC, including histological variants and p16 findings, remain relatively limited in India. This study aimed to describe the demographic and clinicopathological spectrum of 1,000 HNSCC cases, with particular attention to histological variants and p16 expression.

## Materials and methods

Study design and setting

This retrospective observational study was conducted in the Department of Pathology, Government ENT Hospital, Osmania Medical College, Hyderabad, India. The study included patients with histologically confirmed HNSCC diagnosed between January 2023 and December 2024. The unit of analysis was the individual patient with histologically confirmed HNSCC. The study primarily described the demographic and clinicopathological profile of HNSCC, with additional evaluation of histological variants, cervical lymph node involvement, and p16 immunohistochemical expression.

Ethical approval

The Institutional Ethics Committee and Biomedical and Health Research Committee of Osmania Medical College approved the study protocol (approval number: IEC/OMC/M.NO (18)/P-192, dated June 8, 2026). As this was a retrospective study, the analysis was based on previously diagnosed cases, existing clinical records, pathology reports, and archived pathological material. The study required no additional investigations, treatment procedures, specimen collection, or direct patient contact. All identifying information was removed before analysis, and patient confidentiality was maintained throughout. In accordance with the approved retrospective study protocol, separate study-specific informed consent was not applicable.

Case identification and eligibility criteria

A census-based sampling approach was used rather than calculating a predetermined sample size. All consecutive histologically confirmed HNSCC cases diagnosed during the two-year study period were screened for eligibility. A total of 1,000 cases satisfying the study criteria were included in the final analysis.

Eligible cases comprised primary SCCs arising from the oral cavity, oropharynx, nasopharynx, hypopharynx, larynx, and other included mucosal head and neck sites. Cases were excluded when the available material was inadequate for histopathological assessment, paraffin blocks were poorly preserved, or essential clinical or pathological information required for the analysis was unavailable.

Each patient was assigned to one primary anatomical site for analysis. Recurrent tumors and second primary malignancies were identified from the available clinical and pathology records and recorded separately. These events were described as additional clinical outcomes and were not counted as new independent primary cases within the principal 1,000-case analysis.

Data collection and variable definitions

Clinical and pathological information was retrieved from hospital case records, pathology requisition forms, archived histopathology reports, and available laboratory records. Variables included age, sex, primary anatomical site, histological grade, histological variant, cervical lymph node status, recurrence, second primary malignancy, and p16 immunohistochemical expression when tested.

Age was grouped into predefined categories for descriptive analysis. Anatomical sites were coded as mutually exclusive categories so that each patient contributed to only one primary-site group. Histological grade was classified as well differentiated, moderately differentiated, or poorly differentiated. For the principal statistical analysis, cervical lymph node status was recorded from available clinical and pathological documentation as either nodal involvement present or absent.

Some records included information on tobacco use, smokeless tobacco, alcohol consumption, areca nut, and related exposures, but these data were not recorded consistently across the cohort. Reliable definitions of duration, dose, frequency, and combined exposure could therefore not be applied consistently. Consequently, we excluded risk-factor variables from the quantitative analysis rather than treating them as complete exposure data.

Cases with missing information for variables essential to inclusion were excluded during case selection. For variables available only in defined subsets, such as p16 immunohistochemistry, analyses were restricted to cases with recorded results. No statistical imputation of missing values was performed.

Histopathological review and analysis

Archived formalin-fixed, paraffin-embedded tissue blocks and corresponding histological sections were reviewed. Sections of approximately 3-5 µm thickness were stained with hematoxylin and eosin (H&E) and examined by light microscopy.

Conventional SCC was diagnosed on the basis of standard squamous differentiation and was categorized as well differentiated, moderately differentiated, or poorly differentiated according to established histomorphological criteria. Tumors were also reviewed for recognized histological variants using the applicable World Health Organization classification criteria for head and neck tumors.

The histological variants in this series included papillary SCC, verrucous carcinoma, basaloid SCC, spindle cell/sarcomatoid carcinoma, acantholytic SCC, non-keratinizing SCC, and non-keratinizing undifferentiated carcinoma of nasopharyngeal type. Variant classification was based on the characteristic microscopic architecture and cytomorphological features identified on H&E-stained sections.

Ascertainment of cervical lymph node status

Cervical lymph node involvement was determined from the clinical and pathological information available in the medical record and pathology documentation. For the principal analysis examining the relationship between histological grade and nodal spread, lymph node metastasis was coded as a binary outcome: present or absent. When information on the side or distribution of nodal involvement was available, we retained these findings for descriptive assessment.

Selection of cases for p16 immunohistochemistry

p16 immunohistochemistry was performed in a defined subset of 109 cases from the main 1,000-case cohort. This group comprised 59 histological variant cases and 50 conventional SCC cases. The 109 cases were not selected because they were already known to be p16-positive, nor were they randomly sampled from the entire cohort.

Selection was based on histological morphology and the availability of adequate, well-preserved formalin-fixed paraffin-embedded tissue suitable for immunohistochemical staining. Histological variant cases were included when sufficient tissue was available for p16 assessment. The conventional SCC subset included 40 well-differentiated, eight moderately differentiated, and two poorly differentiated tumors selected from archived cases with suitable tissue.

The remaining cases in the overall cohort were not tested because p16 immunohistochemistry was not routinely performed in every HNSCC case during the study period, and appropriate archived tissue was not uniformly available. The p16-tested group should therefore be regarded as an availability-based pathological subset rather than a random or population-representative sample.

p16 immunohistochemistry

Immunohistochemical staining for p16 was performed on selected formalin-fixed, paraffin-embedded tissue sections using a commercially available monoclonal antibody against p16 (Biogenex) and an avidin-biotin-based detection method. Appropriate positive and negative controls were included with each staining run.

Stained sections were evaluated for both nuclear and cytoplasmic immunoreactivity. A case was considered p16 positive when strong, diffuse nuclear and cytoplasmic staining involved more than 70% of tumor cells. Cases showing absent, focal, weak, or patchy staining below this threshold were classified as negative.

Because HPV-specific molecular testing, such as HPV DNA testing, RNA in situ hybridization, or E6/E7 mRNA analysis, was not performed, p16 immunoreactivity was interpreted as a surrogate marker rather than direct confirmation of HPV-driven carcinogenesis. This distinction was particularly relevant for tumors arising outside the oropharynx.

Statistical analysis

Data were entered into Excel (Microsoft Corp., Redmond, WA, USA) and analyzed using SPSS Statistics version 25.0 (IBM Corp. Released 2017. IBM SPSS Statistics for Windows, Version 25.0. Armonk, NY). Descriptive statistics were used for the principal clinicopathological variables. Categorical variables were summarized as frequencies and percentages, while age was presented using predefined age categories.

Associations between categorical variables were assessed using the Pearson chi-square test when expected cell frequencies were adequate. Fisher’s exact test was used when sparse cell counts made the chi-square approximation inappropriate. All statistical tests were two-sided, and a p-value <0.05 was considered statistically significant.

For the analysis of histological grade and cervical lymph node metastasis, lymph node metastasis was defined as the outcome variable, while histological grade was the exposure variable. Well-differentiated SCC served as the reference category. Unadjusted odds ratios with 95% confidence intervals were calculated for comparisons involving moderately differentiated and poorly differentiated SCC. No multivariable regression model was performed.

## Results

Age and sex distribution

Among the 1,000 patients with HNSCC, 832 were men and 168 were women, giving a male-to-female ratio of approximately 4.9:1. The largest proportion of patients was in the 51-60-year age group (29.0%), followed by 61-70 years (22.6%) and 41-50 years (22.0%). Patients aged 31-40 years accounted for 14.0% of the cohort, while 6.0% were aged 21-30 years and 6.4% were older than 70 years. Overall, most cases occurred between 41 and 70 years of age, with a clear male predominance across the age groups.

Anatomical site distribution

Table 1 shows the anatomical distribution of the 1,000 HNSCC cases. The oral tongue was the most frequently involved individual site, accounting for 247 cases (24.7%). This was followed by the supraglottis (162 cases; 16.2%), buccal mucosa (159 cases; 15.9%), and glottis (132 cases; 13.2%). Hypopharyngeal tumors accounted for 110 cases (11.0%). Less frequently involved sites were the alveolus/gingiva (6.4%), oropharynx (5.9%), floor of mouth (3.5%), and nasopharynx (3.2%). When grouped by region, oral cavity tumors formed the largest category, while supraglottic and glottic tumors together represented 29.4% of the cohort.

**Table 1:** Anatomical distribution of HNSCC Each case was assigned to one primary anatomical site.

Anatomical site	Number of cases (n)	Percentage (%)
Oral tongue	247	24.7
Buccal mucosa	159	15.9
Alveolus/gingiva	64	6.4
Floor of mouth	35	3.5
Oropharynx	59	5.9
Supraglottis	162	16.2
Glottis	132	13.2
Hypopharynx	110	11.0
Nasopharynx	32	3.2
Total	1,000	100.0

Histological grade and cervical lymph node metastasis

Of the 1,000 HNSCC cases, 710 (71.0%) were well differentiated, 245 (24.5%) were moderately differentiated, and 45 (4.5%) were poorly differentiated. Cervical lymph node metastasis was identified in 147 patients, corresponding to an overall nodal metastasis rate of 14.7%.

The proportion of patients with nodal metastasis differed significantly across histological grades (χ² = 9.89, df = 2, p = 0.007). Lymph node involvement was present in 94 of 710 well-differentiated tumors (13.2%), 50 of 245 moderately differentiated tumors (20.4%), and three of 45 poorly differentiated tumors (6.7%).

Using well-differentiated SCC as the reference category, moderately differentiated SCC was associated with higher odds of cervical lymph node metastasis (OR = 1.68, 95% CI 1.15-2.45; p = 0.007). Poorly differentiated SCC showed lower observed odds of nodal involvement (OR = 0.47, 95% CI 0.14-1.54), but this difference was not statistically significant (Fisher’s exact p = 0.255) (Table 2). The finding in the poorly differentiated group should be interpreted cautiously because only three patients in this category had nodal metastasis.

**Table 2:** Histological grade and cervical lymph node metastasis in HNSCC Lymph node metastasis percentages were calculated within each histological grade. The overall association between histological grade and cervical lymph node metastasis was significant (Pearson chi-square = 9.89, df = 2, p = 0.007). Odds ratios are unadjusted, with well-differentiated SCC as the reference category. SCC: squamous cell carcinoma, LN: lymph node, OR: odds ratio, CI: confidence interval

Histological grade	Cases (n)	Proportion of cohort (%)	Lymph node metastasis n (%)	Unadjusted OR (95% CI)
Well-differentiated SCC	710	71.0	94 (13.2)	1.00 (reference)
Moderately differentiated SCC	245	24.5	50 (20.4)	1.68 (1.15-2.45)
Poorly differentiated SCC	45	4.5	3 (6.7)	0.47 (0.14-1.54)
Total	1,000	100.0	147 (14.7)	-

p16 expression in 59 histological variant cases

A total of 59 histological variant cases were evaluated by p16 immunohistochemistry. Of these, 32 (54.2%) were p16 positive and 27 (45.8%) were negative.

Papillary SCC was the largest subgroup, comprising 32 cases, of which 28 (87.5%) showed p16 positivity. Among the 13 cases of non-keratinizing undifferentiated carcinoma of nasopharyngeal type, one case was p16 positive. One of six spindle cell/sarcomatoid carcinomas was also positive. All five verrucous carcinomas and the single basaloid SCC were p16 negative. The single acantholytic SCC and the single non-keratinizing SCC were p16 positive (Table 3). Because several histological variants were represented by only one or a few cases, these findings were interpreted descriptively. No firm subtype-specific conclusions were drawn from these small groups.

**Table 3:** p16 expression in histological variants of HNSCC Percentage values are not shown for variants represented by a single case because they do not provide a stable estimate of subtype-specific positivity. SCC: squamous cell carcinoma, HNSCC: head and neck squamous cell carcinoma

Histological variant	Cases (n)	p16 negative	p16 positive	p16 positivity (%)
Papillary SCC	32	4	28	87.5
Acantholytic SCC	1	0	1	-
Basaloid SCC	1	1	0	-
Non-keratinizing undifferentiated carcinoma (nasopharyngeal type)	13	12	1	7.7
Non-keratinizing SCC	1	0	1	-
Spindle cell/sarcomatoid carcinoma	6	5	1	16.7
Verrucous carcinoma	5	5	0	0.0
Total	59	27	32	54.2

p16 expression in 50 selected conventional SCCs

Fifty conventional SCC cases from the main cohort were evaluated by p16 immunohistochemistry. These tumors showed the usual SCC morphology without features of a recognized histological variant. The group comprised 40 well-differentiated, eight moderately differentiated, and two poorly differentiated SCCs.

Overall, 20 of the 50 conventional SCCs (40.0%) were p16 positive, and 30 (60.0%) were negative. Positive staining was observed in 14 of 40 well-differentiated tumors (35.0%), five of eight moderately differentiated tumors (62.5%), and one of two poorly differentiated tumors (50.0%). Because the moderately and particularly the poorly differentiated groups were small, the differences in p16 positivity across grades were interpreted descriptively rather than as evidence of a consistent grade-related pattern.

Representative H&E and p16 immunohistochemical images are shown in Figures 1-3. Figure 1 illustrates conventional SCC, while Figures 2-3 demonstrate the histological variants identified in this series together with their corresponding p16 staining patterns.

**Figure 1:**
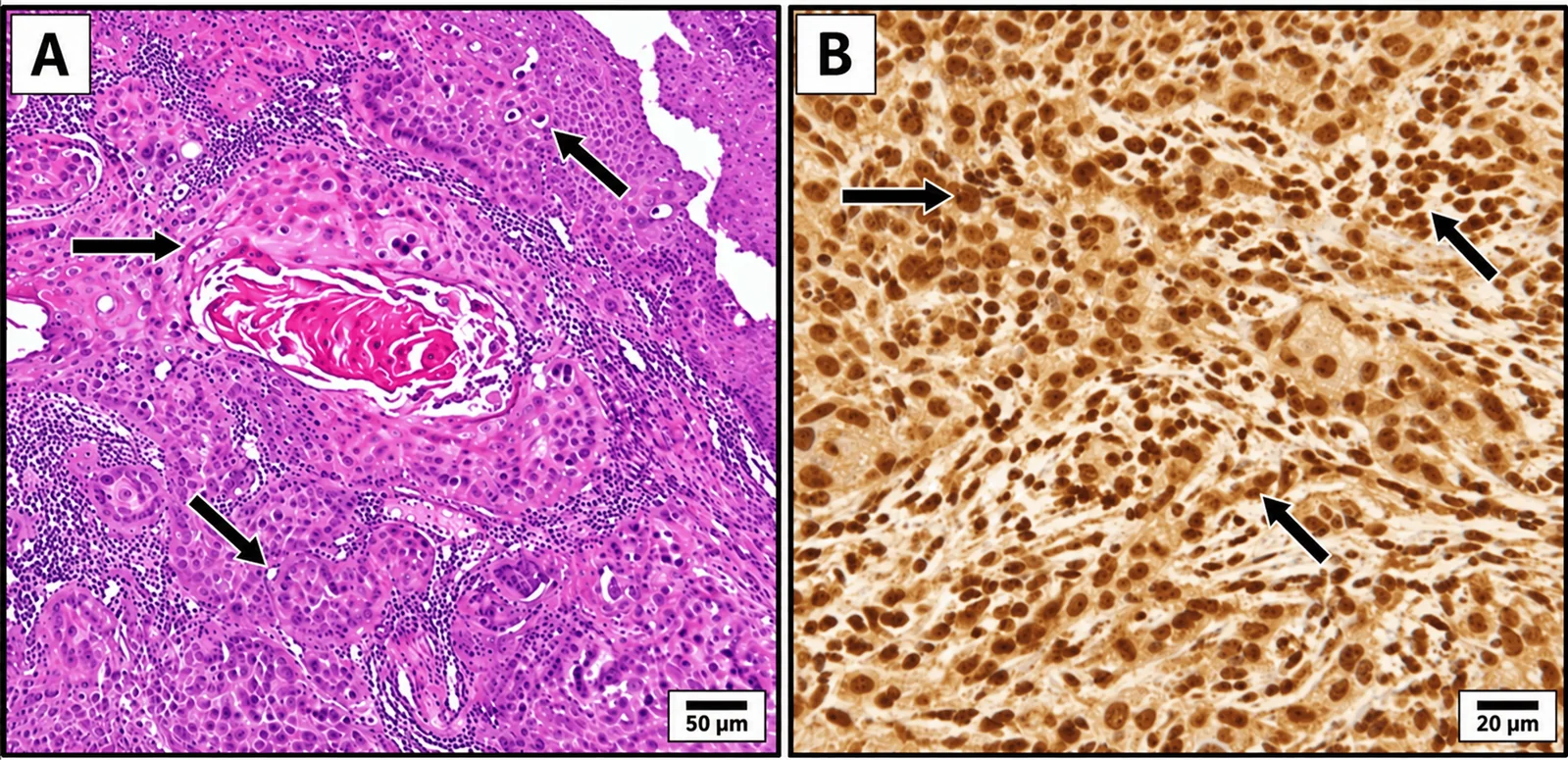
Conventional SCC (A) H&E-stained section showing conventional well-differentiated SCC with keratin pearl formation (black arrow) at 10× magnification. Scale bar: 50 µm. (B) p16 immunohistochemical section from the corresponding tumor showing strong nuclear and cytoplasmic positivity (black arrow) at 40× magnification. Scale bar: 20 µm. H&E: hematoxylin and eosin, HPV: human papillomavirus, IHC: immunohistochemistry, SCC: squamous cell carcinoma

**Figure 2:**
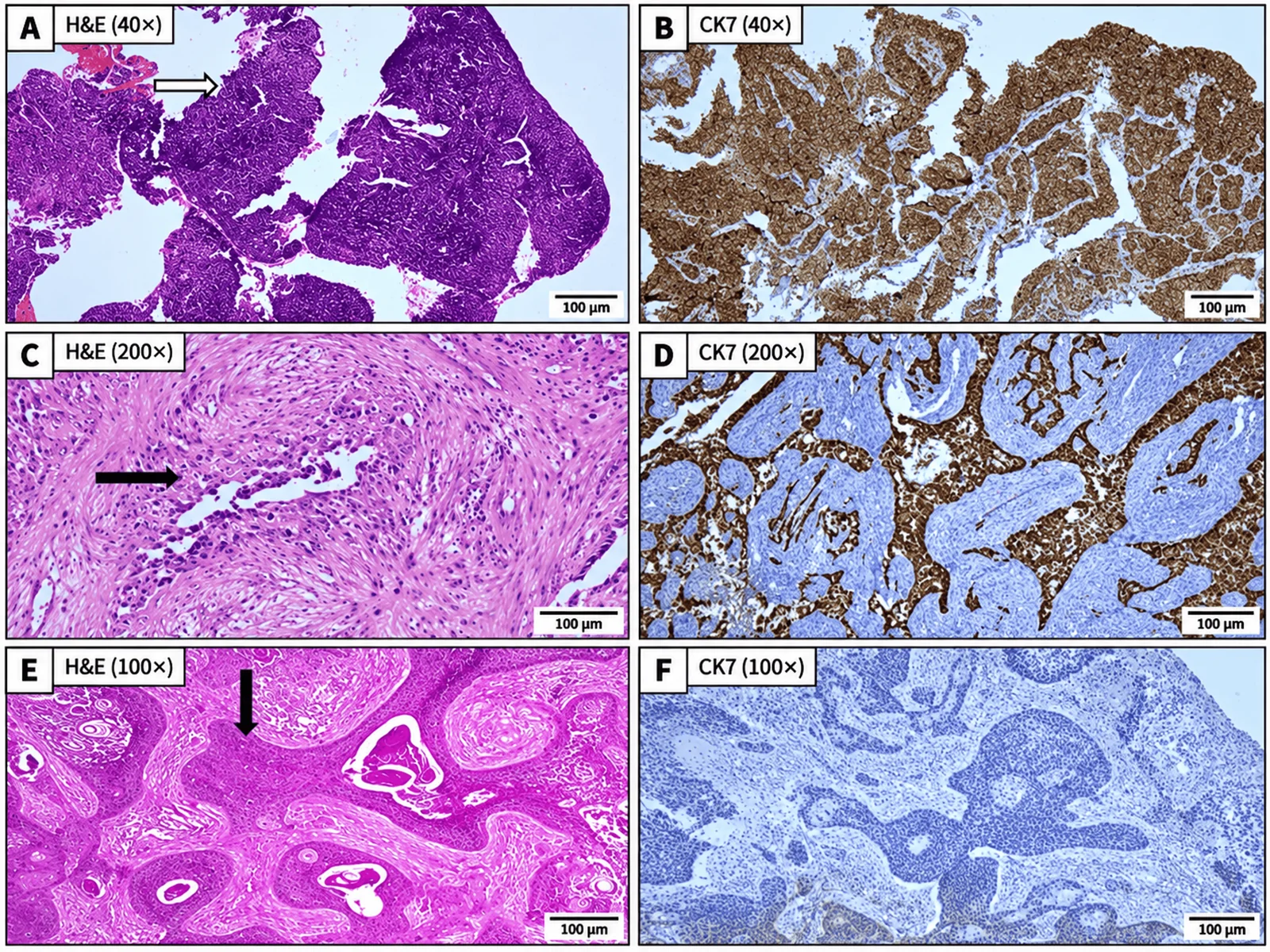
Histomorphological and immunohistochemical features of selected SCC variants (A) H&E-stained section showing papillary architecture with fibrovascular cores (arrow), ×40. (B) Corresponding immunohistochemical section showing diffuse brown staining, ×40. (C) H&E-stained section demonstrating an acantholytic pattern with separation of tumor cells (arrow), ×200. (D) Corresponding immunohistochemical section showing positive staining within the tumor, ×200. (E) H&E-stained section showing basaloid tumor islands with central keratinization (arrow), ×100. (F) Corresponding immunohistochemical section showing absent to weak staining, ×100. Scale bars are shown in each panel H&E: hematoxylin and eosin, IHC: immunohistochemistry, SCC: squamous cell carcinoma, HPV: human papillomavirus

**Figure 3:**
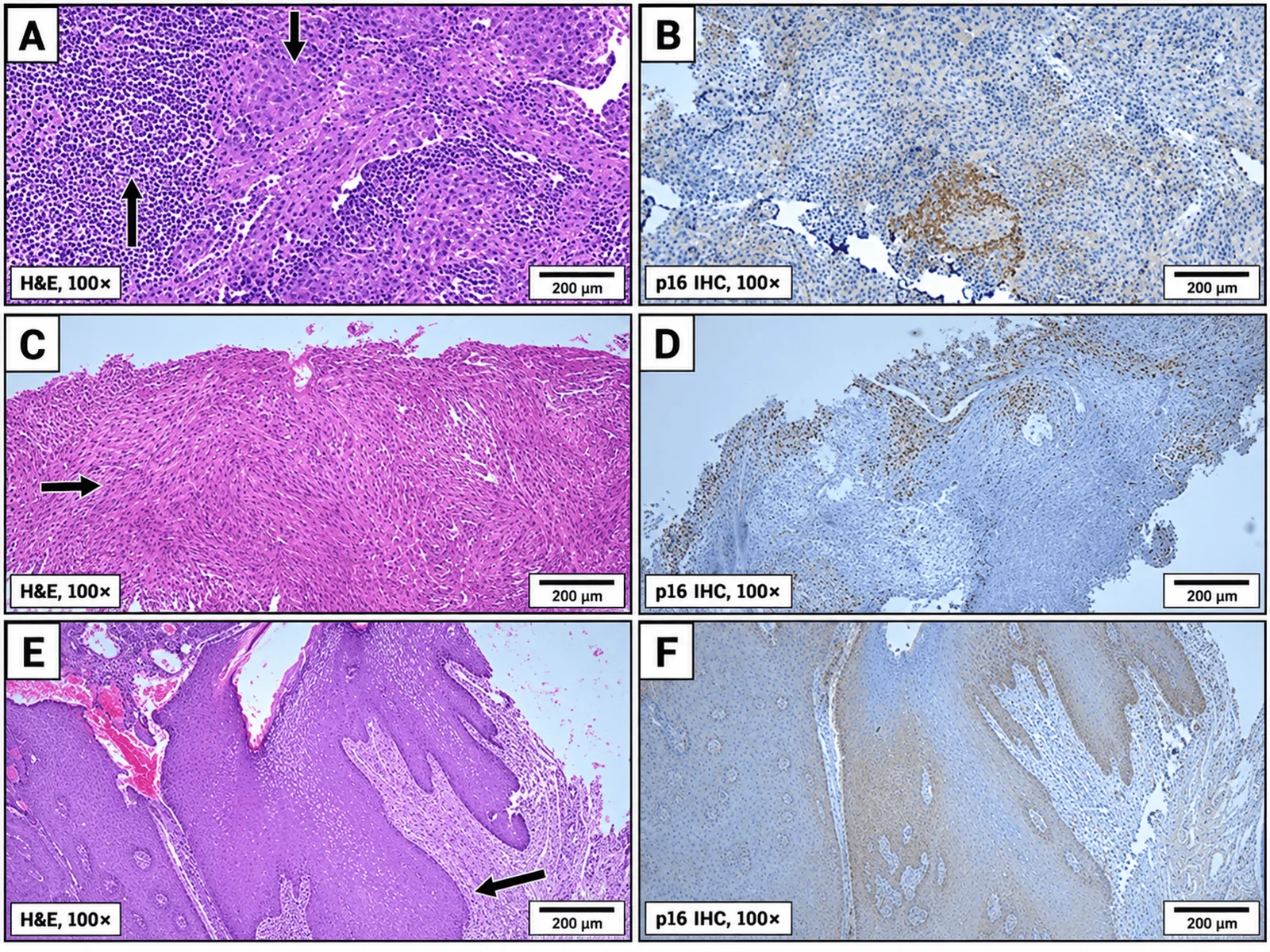
Histomorphological and p16 immunohistochemical features of selected SCC variants (A) H&E-stained section of non-keratinizing undifferentiated SCC of the nasopharynx showing tumor cells and a prominent lymphocytic infiltrate (black arrows), ×100. (B) Corresponding p16 immunohistochemistry showing only focal, weak staining in fewer than 70% of tumor cells, interpreted as negative, ×100. (C) H&E-stained section of spindle cell/sarcomatoid SCC demonstrating spindle-shaped malignant cells (black arrow), ×100. (D) Corresponding p16 immunostain showing patchy weak staining, interpreted as negative, ×100. (E) H&E-stained section of verrucous carcinoma showing broad, well-differentiated epithelial projections with bulbous downgrowth and a pushing margin (black arrow), ×100. (F) Corresponding p16 immunohistochemistry showing weak, patchy staining below the positivity threshold, interpreted as negative, ×100. Scale bars: 200 µm in all panels. SCC: squamous cell carcinoma, H&E: hematoxylin and eosin

p16 expression according to anatomical site in 50 conventional SCCs

Among the 50 conventional SCC cases assessed for p16 expression, 20 (40.0%) were positive. Of these, nine cases arose in the oral cavity, seven in the oropharynx, and four in the larynx. In the full group of 50 tested cases, these represented 18.0%, 14.0%, and 8.0%, respectively (Table 4). Overall, p16 positivity was observed across all anatomical sites, with the oral cavity contributing the largest proportion of positive cases in this cohort.

**Table 4:** p16 expression according to anatomical site in 50 conventional SCCs p16-positive HNSCC cases by anatomical site among 50 conventional SCC cases tested by immunohistochemistry. Percentages were calculated using the total tested cases (n = 50) as the denominator. p16 positivity was interpreted as a surrogate marker. No statistical comparisons were performed. HPV: human papillomavirus, SCC: squamous cell carcinoma

Anatomical site	p16-positive cases	% (n = 50)
	(20 cases)	
Oral cavity	9	18%
Oropharynx	7	14%
Larynx	4	8%
Total	20	40%

p16 expression according to cervical lymph node status

Among the 50 conventional SCC cases evaluated for p16 expression, 39 had no cervical lymph node metastasis, and 11 had nodal involvement. In the node-negative group, 16 of 39 cases (41.0%) were p16 positive, whereas 23 (59.0%) were negative. Among the 11 cases with cervical lymph node metastasis, four (36.4%) were p16 positive and seven (63.6%) were negative. All four p16-positive node-positive cases had bilateral cervical lymph node involvement. The four cases with left-sided and three cases with right-sided nodal metastasis were p16 negative. Overall, p16 positivity was identified in 20 of 50 conventional SCC cases (40.0%) (Table 5).

**Table 5:** p16 expression versus lymph node metastasis Percentages in individual rows were calculated using the total number of cases within the corresponding lymph node category as the denominator. The total row percentage represents overall p16 positivity among the 50 conventional SCC cases. LN: lymph node, SCC: squamous cell carcinoma

Cervical lymph node status	p16 negative, n	p16 positive, n	Total, n	p16 positivity, n (%)
No nodal metastasis	23	16	39	16 (41.0)
Bilateral cervical lymph node metastasis	0	4	4	4 (100.0)
Left cervical lymph node metastasis	4	0	4	0 (0.0)
Right cervical lymph node metastasis	3	0	3	0 (0.0)
Total	30	20	50	20 (40.0)

Overall, p16 positivity was slightly more frequent among node-negative cases (41.0%) than among cases with cervical lymph node metastasis (36.4%).

Second primary tumors and recurrence

A total of 41 patients had either a second primary malignancy or recurrence: 25 had second primary tumors and 16 had recurrences. Among these, the most common prior sites of malignancy were the hypopharynx (nine cases), followed by the glottis (five cases) and the oral cavity (five cases). This distribution highlights the propensity for field cancerization in the upper aerodigestive tract and underscores the importance of vigilant long-term surveillance in patients with HNSCC.

## Discussion

This large single-center series provides a detailed overview of the clinicopathological profile of HNSCC encountered in a tertiary-care hospital in South India. A marked male predominance was observed, and most patients were between 51 and 70 years of age. This age and sex pattern is broadly consistent with reports from the Indian subcontinent, where HNSCC commonly affects middle-aged and older adults. Tobacco, areca nut, and alcohol use are well-recognized contributors to the disease burden in this region [11]. However, these exposures were not analyzed quantitatively in the present study because their documentation in the available records was not sufficiently uniform for reliable comparison.

The oral tongue was the most frequently involved individual site, accounting for 24.7% of cases. Supraglottic and glottic tumors together represented 29.4% of the cohort, while hypopharyngeal tumors accounted for 11.0%. The prominence of oral cavity disease is in keeping with observations from South and Southeast Asia, where oral cancer remains common [12]. Laryngeal SCC also contributed substantially to the present series, which is consistent with previous descriptions of laryngeal cancer as an important component of the HNSCC burden [13]. Hypopharyngeal cancers, although less frequent, remain clinically important because they are often diagnosed at a relatively advanced stage and are associated with poorer outcomes [14].

Well-differentiated SCC was the predominant histological grade, comprising 71.0% of the cases, whereas poorly differentiated SCC accounted for only 4.5%. Cervical lymph node metastasis was identified in 14.7% of the total cohort. The proportion of nodal metastasis differed significantly across histological grades. Moderately differentiated tumors had the highest nodal involvement, occurring in 50 of 245 cases (20.4%), compared with 94 of 710 well-differentiated tumors (13.2%). Compared with well-differentiated SCC, moderately differentiated SCC had higher odds of lymph node metastasis (OR 1.68, 95% CI 1.15-2.45; p=0.007). Only 3 of 45 poorly differentiated tumors showed nodal metastasis, and the difference from well-differentiated SCC was not statistically significant. This apparently lower nodal rate in the poorly differentiated group should be interpreted cautiously because this category had few cases. Histological grade alone is therefore unlikely to capture the full biological behavior of HNSCC, and features such as depth of invasion, lymphovascular invasion, perineural invasion, and tumor-host interface should also be considered during pathological assessment [15,16].

This is particularly relevant in oral SCC, where conventional differentiation grade does not always predict clinical behavior. The worst invasion pattern has emerged as an additional prognostic feature at the invasive front. Broad pushing margins and large cohesive tumor islands are generally associated with a more favorable pattern. In contrast, small infiltrating nests, cords, isolated tumor cells, and separated satellite deposits indicate a more aggressive invasive phenotype. These high-risk patterns have been associated with nodal metastasis, recurrence, and poorer disease-free survival [17].

A range of histological variants was identified in the present study. Papillary SCC was the most frequent variant evaluated by p16 immunohistochemistry. Of the 32 papillary SCC cases, 28 (87.5%) showed positive p16 expression. Previous studies have reported HPV-related changes in a proportion of papillary SCCs, particularly at oropharyngeal and laryngeal sites [18,19]. The present findings therefore support the need for careful morphological recognition of this variant, although p16 positivity alone should not be considered proof of HPV-driven disease.

Among the 13 non-keratinizing undifferentiated carcinomas of nasopharyngeal type, only one case was p16 positive. This is compatible with the well-established predominance of Epstein-Barr virus-related biology in conventional nasopharyngeal carcinoma, although HPV-associated nasopharyngeal carcinomas have also been described [20]. All five verrucous carcinomas and the single basaloid SCC were p16 negative in this series. Verrucous carcinoma is generally regarded as a low-grade, HPV-independent tumor [21,22]. The single acantholytic SCC and the single non-keratinizing SCC were p16 positive, but these isolated observations are not sufficient to infer a stable subtype-specific pattern. For this reason, the p16 findings in very small variant groups were interpreted descriptively rather than comparatively.

p16 immunohistochemistry was also performed in 50 selected conventional SCCs from the main cohort. Twenty of these 50 cases (40.0%) were p16 positive. The conventional SCC subset included 40 well-differentiated, eight moderately differentiated, and two poorly differentiated tumors. Within these grade categories, p16 positivity was seen in 14 of 40 well-differentiated tumors (35.0%), five of eight moderately differentiated tumors (62.5%), and one of two poorly differentiated tumors (50.0%). Although the highest proportion was observed in the moderately differentiated group, the small number of moderately and poorly differentiated cases limits firm conclusions about a relationship between histological grade and p16 expression.

The anatomical distribution of p16-positive conventional SCCs also requires cautious interpretation. Nine of the 20 p16-positive cases arose in the oral cavity, seven in the oropharynx, and four in the larynx. These figures represent the distribution of positive cases within the selected 50-case subset and should not be interpreted as site-specific prevalence estimates. This distinction matters because p16 is an established surrogate marker for HPV-associated carcinogenesis mainly in oropharyngeal SCC, but its specificity is lower at non-oropharyngeal sites. Therefore, the oral cavity and laryngeal p16-positive cases in this series should not be considered definitively HPV-driven without confirmatory molecular testing.

The pattern observed in the present study showed some similarities and differences when compared with the findings reported by Ralli et al. [23]. In both studies, p16 expression varied according to histological grade and anatomical site. Ralli et al. also reported a greater proportion of p16-positive tumors among node-negative than node-positive cases. In the present 50-case conventional SCC subset, 16 p16-positive cases were node-negative, while four p16-positive cases had nodal metastasis. Differences between the two studies may reflect sample size, case selection for immunohistochemistry, tumor-site distribution, and regional variation. Direct comparison of percentages should therefore be approached carefully because the denominators and composition of the study groups were not identical.

Second primary malignancies and recurrences were also observed in this cohort. Twenty-five patients had a second primary tumor, and 16 had recurrence. The occurrence of multiple tumors within the upper aerodigestive tract is consistent with the concept of field cancerization, in which prolonged carcinogenic exposure may produce widespread mucosal alterations and increase the risk of additional primary lesions [24]. Similar patterns of second primary tumors involving the oral cavity, pharynx, and larynx have been described previously [25]. These findings underline the importance of continued surveillance following the initial diagnosis and treatment of HNSCC.

Limitations of the study

Several limitations should be considered when interpreting these findings. First, the retrospective design depended on the completeness and accuracy of existing clinical and pathology records. Some variables, particularly tobacco, alcohol, areca nut, and other exposure histories, were not documented in a sufficiently standardized manner to allow reliable quantitative analysis. Risk-factor associations were therefore not included in the present study.

Second, p16 immunohistochemistry was not performed in all 1,000 cases. The p16-tested subset included 109 cases: 59 histological variants and 50 conventional SCCs. These cases were selected on the basis of the availability and suitability of well-preserved archived tissue for immunohistochemical staining. p16 testing was not routinely performed in every HNSCC case during the study period, and suitable tissue was not uniformly available for the remaining cases. This availability-based selection may have introduced selection bias and limits how well the p16 findings generalize to the entire cohort.

Third, p16 expression was used as a surrogate marker and was not confirmed by HPV-specific molecular methods such as DNA PCR, RNA in situ hybridization, or E6/E7 mRNA testing. Consequently, p16-positive tumors, particularly those arising outside the oropharynx, cannot be assumed to represent HPV-driven carcinogenesis. The findings should therefore be interpreted as patterns of p16 expression rather than direct evidence of HPV infection.

Fourth, several histological variants were represented by only one or a few cases. These small numbers prevented reliable comparison between variant subtypes and made percentage estimates unstable. For this reason, we described findings in sparse categories individually rather than treating them as definitive subtype-specific associations.

Finally, the study did not include detailed longitudinal follow-up, treatment response, disease-free survival, or overall survival. Multivariable modeling was also not performed, so the reported associations are unadjusted and may be influenced by other clinical or pathological factors. Despite these limitations, the large overall sample and broad histological spectrum provide useful regional information on HNSCC encountered in routine tertiary-care practice.

## Conclusions

This large single-center series demonstrates the clinicopathological diversity of HNSCC in an Indian tertiary-care setting. Most cases occurred in middle-aged and older adults, with marked male predominance, and oral tongue and laryngeal tumors were common. Histological grade was significantly associated with cervical lymph node metastasis, with the highest nodal involvement in moderately differentiated tumors. Recognizing histological variants and appropriately assessing p16 expression may provide useful additional information, although p16 positivity should not be considered definitive evidence of HPV-driven disease, particularly outside the oropharynx. Careful pathological evaluation and continued surveillance for nodal disease, recurrence, and second primary malignancies remain important.
